# Levels of Carbohydrate Antigen 125 in Patients With Adult Onset Still Disease: A Case Report

**DOI:** 10.7759/cureus.11194

**Published:** 2020-10-27

**Authors:** Firdevs Ulutaş, Ecem Pars, Veli Çobankara, Serdar Kaymaz, Uğur Karasu

**Affiliations:** 1 Rheumatology, Pamukkale University, Denizli, TUR; 2 Internal Medicine, Pamukkale University, Denizli, TUR

**Keywords:** adult onset still disease, carbohydrate antigen 125, inflammation

## Abstract

Adult onset Still disease (AoSD) is a rare systemic polygenic non-familial autoinflammatory disease. There is no specific biological parameter for diagnosis of AoSD today. This paper presents a case series of three patients with AoSD who had elevated baseline levels of carbohydrate antigen 125 (CA 125). The clinical course of patients was favorable with treatment modalities including steroids and non-steroidal anti-inflammatory drugs. After a comprehensive literature search, it appears that this is the first paper on the association between AoSD and CA 125.

## Introduction

Adult onset Still disease (AoSD) is a rare systemic polygenic non-familial autoinflammatory disease that was defined by Eric Bywaters in 1970 [1]. Its prevalence in Turkey (6.77/100,000) has been reported to be higher than its worldwide prevalence [2]. The Yamaguchi criteria are the most widely used classification criteria, which were first described in 1992 [3]. Although many serum biomarkers have been studied as predictors of disease activity and/or diagnosis, today there is no specific diagnostic parameter for AoSD. AoSD is still a diagnosis of exclusion.

Serum tumor markers are heterogeneous molecules with elevated concentrations in patients with solid tumors. Increased serum concentrations are obtained via cell necrosis, changed expression, or secretion of different molecules. There are several serum tumor markers that are used for diagnosing and monitoring cancer patients in routine clinical practice, including carbohydrate antigen 125 (CA 125). Serum tumor markers can also be found in measurable levels in the plasma of healthy individuals under physiologic and/or inflammatory conditions [4].

Ferritin is the best known biomarker for patients with AoSD. Many cytokines including interleukin-18 (IL-18), tumor necrosis factor (TNF), and interleukin-6 (IL-6) regulate ferritin synthesis through hepcidin synthesis [5]. Among these proinflammatory cytokines, IL-18 has been shown to be more specific than the other cytokines in the diagnosis of AoSD [6]. Higher levels of IL-18 are also correlated with disease activity and inflammatory laboratory results [7]. However, IL-18 has also been shown to contribute to pathophysiological mechanisms in a number of malignancies including gynecological cancers [8]. We present a case series of three patients with AoSD who had elevated baseline levels of CA 125. Their values returned to the normal range after the resolution of inflammation.

## Case presentation

All of the medical records of AoSD patients (n = 61) were screened for laboratory values and presenting manifestations at the diagnosis. All of the patients were diagnosed with AoSD from June 2010 to December 2020 in the Department of Rheumatology, Pamukkale University School of Medicine, Denizli, and all of them fulfilled the classification criteria of Yamaguchi. It was noticed that in three female AoSD patients (n = 3), CA 125 was detected at high titers while diagnosis. The control values of CA 125 after sixth months of treatment were in normal ranges. We aimed to report the changes of CA 125 levels in these three patients. Detailed clinical and laboratory findings of all three patients at diagnosis and after sixth months of treatment are summarized in Table 1. Written consent forms were obtained from all three patients. Ethics committee approval was obtained with date 17/03/2020 and number 06 by Pamukkale University for this paper.

All of the patients were females, and their ages were 55, 41, and 49, respectively. These three patients were hospitalized to investigate the etiology of fever of unknown origin in 2012, in 2015, and in 2018, respectively. The common features of the three patients were fever (body temperature above 39°C), arthralgia, neutrophilic leukocytosis, and increased levels of erythrocyte sedimentation rate (ESR), C-reactive protein (CRP), ferritin, and CA 125. Cervical culture, abdominal magnetic resonance imaging, and Pap smear tests were performed for all patients to exclude infection and gynecological malignancy. Rheumatologic markers including rheumatoid factor (RF), antinuclear antibodies (ANA), and anti-citrullinated protein antibodies were negative for the three patients. All of the three patients fulfilled the Yamaguchi classification criteria as mentioned below [3]. After excluding malignancy, infection, and other rheumatic diseases, fever, arthralgia, skin eruption, sore throat, neutrophilic leukocytosis, and negativity of RF and ANA were defined as positive criteria for the first case. For the second case, fever, arthralgia, elevated liver dysfunction tests, negativity of RF and ANA, and neutrophilic leukocytosis supported the diagnosis. For the third one, fever, arthralgia, skin eruption, neutrophilic leukocytosis, negativity of RF and ANA, and sore throat suggested our diagnosis.

All of the patients were consulted with rheumatologists and ultimately diagnosed with AoSD on an average of five weeks (three weeks, five weeks, and seven weeks retrospectively) after the presenting manifestations and treated with steroids (methyl prednisolone 1 mg/kg/day, tapering to 5 mg/day at the sixth month) and non-steroidal anti-inflammatory drugs. Serum tumor markers were in the normal range after six months of treatment for each patient. Significant clinical improvement was observed in each patient.

Literature search

All papers that were related to tumor markers and AoSD, accessible in full text and written in English, were screened in PubMed databases from 1980 to 2019. The keywords including "tumor markers," "adult onset still disease," and "carbohydrate antigen 125" were used for screening. After a comprehensive literature search, it appears that this is the first paper on the association between AoSD and tumor markers. These three rare cases are presented with a brief review.

This paper is a single center experience, and time period research was done for AoSD patients. In the past 10 years, three AoSD patients have been investigated for CA 125 at diagnosis and after sixth month of treatment, as reported in this paper.

## Discussion

Unlike an underlying unknown etiology, an imbalanced immunity between uncontrolled inflammation and resolution is well known as a pathogenetic mechanism in AoSD. In patients with a predisposing genetic background, the nucleotide-binding oligomerization domain (NOD)-like receptor (NLR) family pyrin domain (PYD)-containing protein 3 (NLRP3) inflammasome and neutrophils activate as a response to danger signals. Increased neutrophils and/or leukocytes and ultimately intensive inflammation lead to tissue damage and clinical findings in autoinflammatory diseases [9]. Markedly increased ferritin is typical for AoSD compared to what is observed in other autoimmune, inflammatory, infectious, or neoplastic diseases [10]. Novak et al. described six typical cases where an extremely high serum ferritin level above 5000 µg/L was seen in all of them [11]. Also, elevated levels of ESR and CRP, the presence of neutrophilia and/or neutrophilic leukocytosis, seronegativity, and elevated serum transaminases contributed to the diagnosis, as in our cases [12]. The interesting point is that all of our patients had higher CA 125 values at the beginning. Today we know that not only malignancies but many inflammatory/infectious diseases and physiological conditions may cause increased levels of tumor markers. CA 125 is a glycoprotein, expressed on the surface of both ovarian cancer cells and healthy cells of mesothelial origin. Immunohistochemical staining of normal cervical tissue in healthy women demonstrated the synthesis and secretion of this tumor marker in the tall columnar cells of the endocervical epithelium [13]. Elevated levels were found in patients with gynecologic tumors, pelvic inflammatory disease, ascites, and tubo-ovarian abscess as well as in healthy women with pregnancy or a normal menstrual cycle due to increased local expression in the area of inflammation [14].

In rheumatology, CA 125 elevation is also related to pleural effusion, ascites, and protein-losing enteropathy in systemic lupus erythematosis patients [15]. Activation of NLRP3 leads to intense production of IL-18 in AoSD patients. The overproduction of IL-18 is specifically related to higher serum ferritin levels and more severe inflammation [16]. Gang et al. have also stated that serum biomarkers including CA 125 and IL-18 are more reliable than risk of malignancy indices for ovarian tumors [17]. Weng et al. reported that serum levels of CA 125 and IL-18 were also detected in endometriosis patients after laparoscopic surgical excision at 12 months [18]. We hypothesize that the elevated levels of CA 125 in our patients may be correlated with IL-18 overproduction, intensive non-specific inflammatory response, and hyperferritinemia. Finally, our patients were not diagnosed with malignancy as expected. Also it is well known that recognized tumor markers such as CA 125 have low sensitivity and specificity in a population of rheumatic patients for screening or diagnosing cancer [19].

A few limitations are present for this paper. Retrospective analysis of a few patients was done. We did not search other tumor markers in these patients. It may be explained that currently none of these tumor markers has been used in screening malignancy. Also no analysis of associated proinflammatory cytokines was done because of the retrospective nature of the analysis.

As a result, the baseline high serum CA 125 levels returned to normal values in the mentioned three patients. None of these patients developed gynecological malignancy in the follow-up. This condition emphasized the low sensitivity and specificity of CA 125 in rheumatic disease such as AoSD.

## Conclusions

AoSD is still a diagnosis of exclusion. The objective of this review was to call attention to higher serum tumor markers in AoSD patients that may be related to inflammation. We should investigate tumor markers in required patients rationally. The authors are not recommending to check CA 125 in patients suspected to have AoSD. Their low sensitivity and specificity in rheumatological diseases should be kept in mind.

## Appendices

**Table 1:** Clinical manifestations and baseline laboratory results of three AoSD patients at diagnosis, and their pre- and post-treatment values of CA 125. ALT: Alanine amino transferase, ALP: alkaline phosphatase, ESR: erythrocyte sedimentation rate, CRP: C-reactive protein, CA 125: carbohydrate antigen 125, +/-: present/absent, AoSD: adult onset Still disease. All of the three patients fulfilled the Yamaguchi classification criteria [3].

	Case 1	Case 2	Case 3
Age	55	41	49
Gender	Female	Female	Female
Fever	+	+	+
Skin eruption	+	-	+
Arthralgia/arthritis	+	+	+
Sore throat	+	-	+
White blood cells (K/µL)	20900	13600	12700
Neutrophil count (K/µL)	19900	10100	10400
Rheumatoid factor	Negative	Negative	Negative
Antinuclear antibody	Negative	Negative	Negative
ALT (IU/L)	14	108	35
ALP (IU/L)	75	153	69
Ferritin (ng/mL)	3289	4000	445
ESR (mm/h)	125	62	62
CRP (mg/dL)	214	19	41
CA 125 U/mL (0-35) (at diagnosis)	129	199	73
CA 125 U/mL (0-35) (after treatment)	6.75	12	6.75
